# Integrating Coalitions, Strategic Planning, and Road Map Implementation in Alzheimer’s Disease and Related Dementias Care: A Case Study from Mississippi

**DOI:** 10.1002/alz.088063

**Published:** 2025-01-09

**Authors:** Kina L. White, Sai Kurmana

**Affiliations:** ^1^ Mississippi State Department of Health, Jackson, MS USA

## Abstract

**Background:**

With Alzheimer’s Disease and Related Dementias (ADRD) posing significant public health challenges globally, effective strategies that encompass policy, practice, and community engagement are essential. The Mississippi State Department of Health (MSDH) has implemented an integrated approach to ADRD, focusing on three critical themes: Coalitions and Collaborations, ADRD Strategic Plans, and Healthy Brain Initiative (HBI) Road Map Implementation. This case study provides valuable insights into a comprehensive public health approach towards ADRD.

**Method:**

Recognizing the power of collective action, MSDH facilitated the expansion of the Alzheimer’s State Coalition. This coalition brought together diverse stakeholders, including healthcare providers, policy experts, caregivers, and community organizations. Our analysis highlights the coalition’s role in mobilizing resources, fostering community engagement, and influencing policy decisions. By evaluating the coalition’s structure, engagement strategies, and impact on ADRD initiatives, we provide insights into the effectiveness of collaborative approaches in addressing complex public health challenges. We discuss the expansion and implementation of Mississippi’s 2020‐2025 Strategic Plan for ADRD, which integrated essential components of dementia risk reduction, early diagnosis, management of comorbidities and caregiver support. Our evaluation focuses on the plan’s alignment with national priorities, its adaptability to emerging challenges, and its role in guiding statewide ADRD efforts.

**Result:**

The implementation of the CDC’s HBI Road Map within the MS ADRDP offers a model for integrating national guidelines into state‐level ADRD programs. We analyze how the MS ADRDP incorporated HBI Road Map actions into its strategic planning and operational activities. This assessment includes a review of successes and challenges in implementation, particularly in areas that address Alzheimer’s Disease and Related Dementias, reflecting the broader goals of the CDC BOLD program.

**Conclusion:**

The Mississippi case study underscores the effectiveness of an integrated approach in addressing ADRD. Our findings highlight the significance of collaborative efforts in coalition building, strategic planning, and national guideline implementation. These elements collectively enhance the state’s capacity to manage ADRD challenges effectively. This study offers a scalable model for other regions and countries seeking to develop comprehensive, collaborative, and strategic approaches in ADRD public health policy and practice.

